# Magnitude of dual contraceptive method utilization and the associated factors among women on antiretroviral treatment in Wolaita zone, Southern Ethiopia

**DOI:** 10.1016/j.heliyon.2022.e09595

**Published:** 2022-05-31

**Authors:** Dereje Haile, Berhanu Lagebo

**Affiliations:** a Reproductive Health and Nutrition Department, School of Public Health, College of Medicine and Health Science, Wolaita Sodo University, Sodo, Ethiopia; b Wolaita Zone Health Department, Wolaita Sodo, Ethiopia

**Keywords:** Dual contraceptive, Sexually transmitted infection, Unwanted pregnancy

## Abstract

**Background:**

Expanding the contraceptive options based on desires of families and personal context is critical to address the needs of users. For instance, dual contraceptive methods were recommended for people with the human immunodeficiency virus (HIV) patients to prevent HIV transmission, other sexually transmitted infections (STIs), and unintended pregnancies. Disclosure is one of the strategies to reduce the stigma related to HIV and use of contraception clandestinely. However, there is a dearth of evidence regarding the magnitude of and the factors affecting dual contraceptive method utilization among sexually active women on antiretroviral treatment (ART).There is limited evidence regarding the association between disclosure to community and dual contraceptive utilization. Thus, the aim of this study is to assess the magnitude of dual contraceptive utilization and its associated factors among ART patients in this study area.

**Objective:**

the primary objective of this study was to assess the prevalence of the dual contraceptive use.

**Methods:**

A facility-based cross-sectional study design was employed to collect data from a total of 556 respondents by using a semi-structured questionnaire. Data were entered in EpiData version 3.1 and exported to SPSS version 20 for analysis. Binary logistic regression was applied to determine the magnitude of dual contraceptive utilization and the factors associated with it. Variables with p-value<0.25 were considered as candidate for multivariable logistic regression. In multivariate logistic regression, variables with a *p*-value < 0.05 were reported to be statistically significant.

**Result:**

The magnitude of dual contraceptive utilization was 28.6% (95%CI: 24.8, 32.4). HIV sero-status disclosure to community (AOR:7.1 (95%CI: 4.8,10.2)), disclosure to sexual partners (AOR:3.1 (95%CI:1.2,7.8)), sexual activity (AOR: 4.7 (95%CI: 2.5, 10.0)), fertility desire (AOR:4.3 (95%CI:2.4,7.5)), history of STI (AOR: 3.2 (95%CI: 1.6, 6.6)), partners’ sero-HIV status (AOR:3.7 (95%CI:1.7,8.1)), and discussion with sexual partners about dual contraceptive methods (AOR:5.8 (95%CI:2.2,8.5)) were significantly associated with dual contraceptive utilization.

**Conclusion:**

This study found that a substantial number of mothers did not use dual contraceptive methods. Disclosure to community increases the use of dual contraceptive methods. Integrating the family planning with STI and ART care, strengthening the partner involvement during posttest counseling is recommended to increase the uptake of dual contraceptive methods utilization. Moreover, encouraging PLWHIV to disclose their HIV status to the community should be advocated.

## 1 Introduction

Worldwide, there were approximately 37.9 million people living with HIV by the end of 2018; however, over two-thirds of all the people living with HIV live in the African region (25.7 million); among them, 1.7 million were children [1].Sub-Saharan African countries including Ethiopia account for the majority of the HIV cases and bear the burden of its consequences such as economic, social, and health consequences [2].

Ethiopia is one of the 25 countries with the highest numbers of new HIV infections that were selected for the Global HIV Prevention Coalition [3]. The estimated adult prevalence was 0.9% and a large number of people were living with the virus, 23 000 people were newly infected with HIV,.and 11 000 people had died from an AIDS-related illness [4].

The rate of HIV transmission also varied globally. According to the 2017 Global information and education on HIV and AIDS report, the overall transmission rate of HIV among children in Ethiopia was 15.4%, which was higher than that in Indonesia, Angola, and Ghana [5].

Since 2010, the progress of access to HAART has been increasing, which is very important for improving the quality of life of people living with HIV. Despite this, unintended pregnancy, contraction of new strains of HIV, and pediatric HIV have become challenging issue [6].It is worth mentioning that, by the end of 2018, more than half (55.9%) of the pregnancies among PLWHA were unintended, which was mainly due to poor contraceptive utilization [7, 8].

In sub-Saharan Africa, increasing the contraceptive prevalence rate (CPR) has been estimated to reduce the proportion of infants infected with HIV by 35–55% through reduction in primary HIV infection and unintended pregnancies in HIV-infected women [9]. Dual contraceptive method utilization can reduce the risks of both unintended pregnancy and sexually transmitted infections (STIs) including HIV [10, 11]. Only condom utilization has a dual effect; however, its protection is partial [11]. Moreover, the efficacy of other modern contraceptive methods can be reduced by some ART drugs because of drug interactions, which necessitates the use of condoms to compensate for this gap [6, 12].

Despite its advantage, a number of studies found a low level of magnitude of the utilization of dual contraception method [13, 14]. Studies from various parts of Ethiopia revealed that g factors that affect the use of dual contraceptive methods are HIV status disclosure, educational status, partner's discussion on family planning, and so on.

Disclosure of positive HIV status by PLHIV to their sexual partners is important in reducing HIV/AIDS transmission. This is because it may prompt the partner of an HIV-infected individual to seek HIV testing and take up other interventions such as condom use [15]. Moreover, it increases the chances of obtaining support for taking one's medications and attending clinic appointments [16].

However, even if the magnitude of disclosures to sexual partners is increasing and its association with dual contraceptive method utilization is well known, disclosure to community and its association with contraceptive utilization is limited. Moreover, there is scarcity of evidence regarding the magnitude of and factors affecting dual contraceptive method utilization among sexually active women on ART. Therefore, the aim of this study will be to assess the prevalence of dual contraception method utilization and the associated factors among women on ART at health facilities in Wolaita Zone, SNNPR Ethiopia.

## 2 Methods and materials

### 2.1 Study setting and design

The study was conducted at Wolaita Zone, which is situated in southern nation nationality and people's region and one of 13 zones, located 380 km south of Addis Ababa (the capital of Ethiopia) and 157 km south of Hawassa (the capital city of SNNPR). Wolaita Zone is further divided into 17woreds, 5 town administration and 355 Keble (smallest administrative unit).

According to the 2012 Woreda-based plan, Wolaita Zone has a total population of 2067163, of which 1,151,193 are males and 915,970 are females. Women of child-bearing age are 142, 147. There are 7 hospitals, 68 health centers, and 342 health posts. There are 18 ART sites in the zone. The total number of PLHIV is 3,947. There are 2,132 women in the reproductive age group receiving ART. An institution-based cross-sectional study design was employed.

### 2.2 Population

All women on antiretroviral treatment (ART) from the 18 health facilities of Wolaita zone were the source population. All randomly selected women on ART from the selected six health facilities of Wolaita Zone were the study population.

### 2.3 Eligibility criteria

All women on ART in the 18- to 49-year age group at the time of interview were included in this study, while women on ART who were severely ill and transfer out were excluded from this study.

### 2.4 Sample size determination and sampling procedure

#### 2.4.1 Sample size determination

The sample size for the first objective is estimated using the single population proportion formula by considering a confidence level of 95%, a margin of error of 5%, a proportion of 19.8% which is taken from a study conducted by Meseret Woldemariamin 2015 [17].

$$\begin{array}{l} \text{n}=\frac{\left(\text{Z}\alpha \text{/}2\right)\text{p}\left(1-\text{p}\right)}{{\text{d}}^{2}}\\ \;=\frac{\left(1.96\right)20.198\left(1-0.198\right)}{{\left(0.05\right)}^{2}}\\ \;=243\text{ ​} \end{array}$$

By considering 10% (24) contingency for the non-response rate, the sample size was 267.

Thus, the calculated sample size for the first objective was 267 participants.

The sample size for the second objective was calculated by using factors associated with dual contraceptive utilization such as HIV status disclosure to partners and discussion with partners regarding dual contraceptive utilization [14], and pregnancy since HIV diagnosis [13]. As shown in Table 1, the pregnancy since HIV diagnosis category has the largest sample size of 376. The final sample size after adding 10% non-response rate and multiplying it by 2 (design effect) will be 752. After applying the correction formula, it was 556.

**Table 1:** Sample size calculation for the second objective.

Variable	CI	Power	Percentage outcome among Exposed	AOR	Sample size	Non-response rate 10%	Total sample size	References
Discussion with partner about dual contraceptive use	95%	80%	28.57%	7.84	42	4	46	[14]
Pregnancy since HIV diagnosis	95%	80%	20.5%	2.05	342	34	376	[13]

The final sample size was 556.

#### 2.4.2 Sampling procedure

In wolaita zone, southern Ethiopia, there are 18 health facilities that provide ART to PLWHIV. Of 18, a total of 6 health facilities were selected by the lottery method. To allocate clients proportionally, ART registry of the patient load in each institution was used. The previous average daily client flow was used to estimate average number of clients who visited the ART units daily during the data collection period. Systematic random sampling was performed to select the study subjects from the selected ART sites. The exit interview was conducted by using the interval (K) for each institution which was calculated for each institution according to proportional allocation. The first participants at each health institutions were selected by using the lottery method and every 2^nd^, 3^rd^, 3^rd^, 3^rd^, 4^th^, and 5^th^ individuals were recruited for health facilities 1 to six respectively.

### 2.5 Variables

#### 2.5.1 Dependent variable

Dual contraceptive method utilization.

#### 2.5.2 Independent variables

Socio-demographic characteristics: Current age, education, marital status, age at first marriage, religion, residence, and ethnicity.

### 2.6 Socio-economic factors: income and occupation

Individual related factors: Sexual activity, disclosure of the HIV status to the partner, discussion on dual method utilization with the partner, having biological children, pregnancy since HIV diagnosis, future fertility desire, having information about dual family planning methods, and HIV status of the partner.

### 2.7 Data collection procedure

The data were collected by two diploma-holding nurses and one BSC nurse supervisor from six selected ART service providing health facilities in Wolaita Zone by using face-to-face interviewer-administered questionnaires. Structured questionnaires were used, which were adopted by reviewing different literatures [17, 18, 19, 20, 21].

### 2.8 Operational definition

Dual Contraceptive: Utilization of any hormonal or permanent modern contraceptive method along with male or female condoms [22, 23].

Current use of the dual method: Sexually active respondents utilizing reversible or irreversible methods of contraception along with male or female condoms during sexual intercourse.

Disclosure of the HIV status: Disclosing HIV-positive status to the partner and other relatives.

Knowledge of the dual method: We asked the clients eight knowledge-related questions and calculated the mean value. Then we categorized clients as knowledgeable if they answered 4 and above questions correctly, unless otherwise categorized as poor knowledge.

### 2.9 Data management

Data entry was done using Epi-Data version 3.1 after which the exported data were analyzed using SPSS version 23. The collected data were checked for completeness and consistency on a daily basis. Data qualitywas ensured by giving appropriate training to data collectors. Moreover, close supervision was also done during data collection for ensuring completeness and consistency. Double data entry was done to check accuracy. Data cleaning was performed by running a frequency distribution for each variable using SPSS version 23.

### 2.10 Data quality assurance

Data quality was maintained by providing 2 days of training on the topics of tools and data collection procedures. The training was focused on the objectives of the study, the introduction of the questionnaire, and the procedure of interviewing study participants. Pretesting was done in 5% of the total sample out of the study area. Supervision and follow-up were carried out throughout the data collection.

The quality of data was controlled at different levels for completeness and consistency by the data collector, supervisors, and investigators and then the data were entered and cleaned using EPI Data statistical software.

### 2.11 Data analysis

Data were entered into Epi-data software version 3.1 and then were exported to SPSS version 20 statistical package for analysis. Descriptive statistics was performed and summarized using tables, frequencies, graphs, median, proportion, and the inter-quartile range.

The logistic regression model was fitted to identify the factors associated with dual contraceptive method utilization after checking the assumption. The model fitness was checked using the Hosmer and Lemeshow goodness of fit test (*p*-value < 0.05), multi-collinearity was checked by observing whether standard errors were inflated or not. Initially, bivariate logistic regression analysis was performed sequentially between the dependent and each of the independent variables. Variables with a *p* value of <0.25 in bivariate logistic regression will be considered as potential candidates for multivariate logistic regression analysis to control confounding in regression models and were entered by using backward stepwise regression. An association between the outcome variable and the independent variables was observed by using both the adjusted odds ratio and its 95% CI, and variables having a *p* value of less than 0.05 in the multivariate logistic regression model were considered as statistically significant. The reliability analysis indicated the Cronbach's alpha = 0.82.

### 2.12 Ethical consideration

Letter of permission to conduct the study was obtained from the College of Health Sciences and medicine, School of Public Health, Wolaita Sodo University to the Wolaita zone health department. A formal permission letter was written to each selected health facility from the Wolaita zonal health office. Written consent of the respondent was received by communicating the purpose of research before recruitment. We have confirmed that the ethical clearance was in accordance with the declaration of Helsinki.

## 3 Results

### 3.1 Socio-demographic characteristics of women on ART in wolaita zone, southern Ethiopia

Of the 556 sampled subjects, 545 had participated, which yields a response rate of 98.0%. The majority, 247 (45.3%), of the respondents were in the age group 25–34 years, 295 (54.1%) were married, 142 (26.1%) participants had secondary-level education, and 352 (64.7%) resided in urban areas. With regard to age at first marriage, nearly one-fifth of the study respondents, 108 (19.8%), got married before 18 years (Table 2).

**Table 2:** Socio-demographic characteristics of women on ART in wolaita zone, southern Ethiopia.

Respondent characteristics		N	(%)
Age (n = 545)	15–24	70	12.8
	25–34	247	45.3
	≥35	228	41.8
Marital status (n = 545)	Married	295	54.1
	Single	65	11.9
	Divorced	103	18.9
	Widowed	82	15
Duration of marriage (n = 487)	0–5 years	95	19.5
	6–10 years	246	50.5
	>10 years	146	30
Maternal educational status (n = 545)	Unable to read or write	131	24
	Primary	129	23.7
	Secondary	142	26.1
	College/diploma	85	15.6
	Degree and above	58	10.6
Residence (n = 545)	Rural	193	35.3
	Urban	352	64.7
Employment status (n = 545)	Housewife	144	26.4
	Merchants	103	18.9
	Daily laborer	216	39.6
	Government workers	82	15
Religion (n = 545)	Catholic	56	10.3
	Muslim	42	7.7
	Orthodox	206	37.8
	Protestants	241	44.2
Age at first marriage (n = 545)	<18	108	19.8
	18–24	151	27.7
	25–34	234	42.9
	≥35	52	9.5
Average monthly income (n = 545)	<500	74	13.6
	500–1000	281	51.6
	1001–1500	43	7.9
	>1500	147	27

### 3.2 Reproductive characteristics of women on ART in Wolaita zone, southern Ethiopia

Among the participants, the majority, 361 (66.2%), were sexually active. Regarding fertility desire, 327 (60%) participants did not desire fertility. The number of respondents having 3–4 children was 243 (43.7%). However, of the total participants, only 41 (7.5%) had no child. Of the total participants, 72 (13.2%) had a history of STI (Table 3).

**Table 3:** Reproductive history of women on ART in wolaita zone, southern Ethiopia.

Respondents characteristics		N	(%)
Sexually active (n = 545)	Yes	361	66.2
	No	184	33.8
Fertility desire (n = 545)	Yes	218	40
	No	327	60
Pregnancy since HIV positive (n = 545)	Yes	127	23.3
	No	418	76.7
History of STI (n = 545)	Yes	72	13.2
	No	473	86.8
Parity (n = 545)	No child	41	7.5
	1–2	94	17.2
	3–4	243	44.6
	≥5	167	30.6

### 3.3 Risk prevention behaviors, access to information on dual contraceptive methods, and related medical factors

Among the participants, the majority, 514 (94.3%), had heard about family planning methods. With regard to the types of contraception, the majority, 215 (41.8%) had heard about inject able methods, whereas only 4 (0.8%) had heard about tuba ligation. Majority of the study participants had not disclosed their HIV sero-status to the community 471 (86.4%). More than half of the participants, 287 (55.9%), had obtained contraception from f/p OPD. Out of all the respondents, more than two thirds, 351 (68.3%), had heard about dual family planning methods, 263 (46.1%) had discussed with partners, 456 (83.7%) had disclosed their HIV sero-status, 342 (62.8%) participants were in WHO stage one, and 306 (56.1%) participants had CD4 cell counts of >500 in the last six months (Table 4).

**Table 4:** Risk prevention behaviors, access to information on dual contraceptive methods, and related medical factors among the study participants.

Respondent characteristics		N	(%)
Ever heard about any f/p methods (n = 545)	Yes	514	94.3
	No	31	5.7
Types of f/p (n = 514)	Injectables	215	41.8
	Pills	93	18.1
	Male condom	68	13.2
	Implants	111	21.6
	IUCD	23	4.5
	Tuba ligation	4	0.8
Source of family planning (n = 514)	ART clinic	190	37
	F/P OPD	287	55.9
	Other OPD	24	4.6
	Pharmacy room	13	2.5
Ever heard about dual f/p methods (n = 514)	Yes	351	68.3
	No	163	31.7
Source of information about dual f/p methods (n = 514)	Mass media	115	22.4
	Health professionals	386	75.1
	Friends	13	2.5
Disclosure to community	No	471	86.4
	Yes	74	13.6
HIV status disclosure to sexual partners (n = 545)	Yes	456	83.7
	No	89	16.3
Discussion with husbands (n = 514)	Yes	263	46.1
	No	251	53.9
HIV sero-status of current sexual partners (n = 383)	Positive	252	65.8
	Negative	41	10.7
	Unknown	90	23.5
CD4 cell count in the last six months (n = 545)	>500	306	56.1
	350–500	156	28.6
	<350	83	15.2
Current WHO stages	One	342	62.8
	Two	146	26.8
	Three	57	10.5

### 3.4 Utilization of dual contraceptive methods

**Figure 1:**
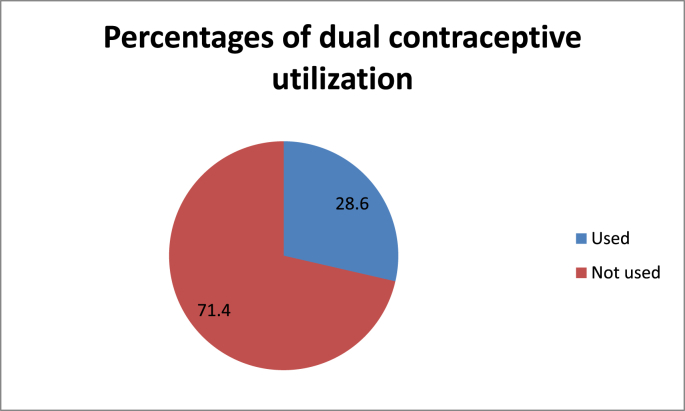
The percentages of dual contraceptive utilization among HIV patients on ART at health facilities in Wolaita Zone.

The magnitude of dual contraceptive utilization in this study was 28.6% (95%CI: 24.8, 32.4). The majority of the study participants had used condoms plus injectables (26.6%), followed by condoms plus implants (15.4%), condoms plus pills (7.3%), and condoms plus IUCDs (2%) (Figure 1).

### 3.5 Determinants of dual contraceptive method utilization

The current study found that in bivariable logistic regression, marital status, residence, average monthly income, parity, being sexually active, HIV sero-status disclosure, history of STIs, discussion with sexual partners about dual contraceptive methods, knowledge about the HIV status of sexual partners, fertility desire, information regarding about dual family planning, and pregnancy since HIV positive were identified as significant. In multivariable logistic regression, disclosure to community, HIV sero-status disclosure, history of STIs, discussion with sexual partners about dual contraceptive methods, knowledge regarding the HIV status of sexual partners, fertility desire, and being sexually active were identified as predictors of dual contraceptive utilization.

The odds of utilizing dual contraceptive methods were 7 times higher for participants who had disclosed their HIV sero-status to community than that for their counterparts.

The odds of utilizing dual contraceptive methods were 4.7 times higher for those who were sexually active than that for their counterparts (AOR: 4.7 (95%CI: 2.5, 10.0)). The likelihood of utilizing dual methods were 4.3 times higher for those study participants who had no fertility desire compared with that for those who had fertility desire (AOR:4.3 (95%CI:2.4,7.5)). Participants who had no a history of STIs were significantly more likely to use dual contraception methods compared with those who had no history of STIs (AOR: 3.2 (95%CI: 1.6, 6.6)). Study participants whose sexual partner was HIV positive were 3.7 times more likely to use dual contraceptive methods compared with those participants whose sexual partner was HIV negative (AOR:3.7 (95%CI:1.7,8.1)). Disclosure of the HIV sero-status was significantly associated with dual contraceptive utilization. The likelihood of utilizing dual contraceptive methods was 3.1 times higher for study participants who disclosed their HIV sero-status to sexual partners when compared with their counterparts (AOR:3.1 (95%CI:1.2,7.8)). Discussion with sexual partners about dual contraceptive methods increases its utilization level by 5.2 times when compared with study participants who had no discussion with sexual partners (AOR:5.8 (95%CI:2.2,8.5)) (Table 5).

**Table 5:** Bivariable and multivariable logistic regression analysis of the predictors of dual contraceptive utilization.

Respondent characteristics		Dual contraception method used		*p*-value	COR (95%CI)	AOR (95%CI)
		No	Yes			
Marital status (n = 545)	Married	196 (66.4)	99 (33.6)	0.028	1.9 (1.1,3.5)	0.6 (0.3,1.4)
	Single	48 (73.8)	17 (26.2)	0.440	1.3 (0.6,2.9)	0.6 (0.2,1.7)
	Divorced	80 (77.7)	23 (22.3)	0.793	1.1 (0.5,2.2)	0.9 (0.4,2.4)
	Widowed	65 (79.3)	17 (20.7)		1	1
Residence (n = 545)	Rural	144 (74.6)	49 (25.4)		1	1
	Urban	245 (66.6)	107 (30.4)	0.217	1.3 (0.8,1.9)	0.8 (0.4,1.4)
Average monthly income (n = 545)	<500	58 (82.9)	16 (21.6)		1	1
	500–1000	214 (78.1)	67 (23.6)	0.688	1.1 (0.6,2.1)	0.9 (0.4,2.0)
	1001–1500	27 (62.8)	16 (37.2)	0.071	2.1 (0.9,4.8)	1.2 (0.4,3.4)
	>1500	90 (57)	57 (39.8)	0.012	2.3 (1.2,4.4)	1.6 (0.7,4.0)
Parity	No child	27 (65.9)	14 (34.1)	0.224	1.6 (0.7,3.3)	2.0 (0.8,5.3)
	1–2	69 (73.4)	25 (26.6)	0.219	1.4 (0.8,2.5)	1.9 (0.8,4.5)
	3–4	174 (71.6)	69 (28.4)	0.345	1.2 (0.8,2.0)	1.5 (0.8,2.6)
	≥5	119 (71.3)	48 (28.7)		1	1
Sexually active	Yes	224 (62)	137 (38)	0.000	5.3 (3.1,8.9)	4.7 (2.5,10.0)
	No	165 (89.7)	19 (10.3)		1	1
Disclosed HIV sero-status to community	No	368 (78.1)	103 (21.9)			
	Yes	21 (28.4)	53 (71.6)	0.000	9.0 (5.2,15.6)	7.1 (4.8,10.2)∗∗
Fertility desire	Yes	170 (78)	48 (22)		1	
	No	219 (67)	108 (33)	0.006	1.7 (1.2,2.6)	4.3 (2.4,7.5)∗∗
History of STIs	Yes	58 (80.6)	14 (19.4)		1	1
	No	331 (70)	142 (30)	0.067	1.7 (0.9,3.3)	3.2 (1.6,6.6)∗∗
Pregnancy since HIV positive	Yes	78 (61.4)	49 (38.6)		1	1
	No	311 (74.4)	107 (25.6)	0.005	1.8 (1.2,2.7)	1.5 (0.9,2.6)
Ever heard about dual f/p	Yes	205 (58.4)	145 (41.6)	0.812	1.05 (0.7,1.6)	
	No	114 (69.9)	50 (30.1)		1	
Disclosed HIV sero-status to sexual partners	Yes	317 (69.5)	139 (30.5)	0.032	1.8 (1.1,3.3)	3.1 (1.2,7.8)∗∗
	No	72 (80.9)	17 (19.1)		1	1
Discussion with sexual partners on dual f/p	No	200 (79.7)	51 (20.3)		1	1
	Yes	127 (49.3)	136 (51.7)	0.001	4.0 (2.8,6.0)	5.8 (2.2,8.5)∗∗
Partners' HIV status	Positive	146 (57.9)	106 (42.1)	0.002	4.2 (1.7,10.4)	3.7 (1.7,8.1)∗∗
	Unknown	66 (73.3)	24 (26.7)	0.134	2.1 (0.8,5.7)	1.7 (0.8,3.9)
	Negative	35 (85.4)	6 (14.6)		1	1

∗∗ indicates significance at the 5% level of significance in multivariable logistic regression.

## 4 Discussion

The current study revealed that the magnitude of dual contraceptive utilization was 28.6% (95%CI: 24.8, 32.4). This finding was in line with the findings reported in studies conducted in Thailand (29.6%) [24] and Southeastern Nigeria (25.1%) [25]. Similarly, the findings in other studies are as follows: Gimbie town (30%) [26], Fitche town (32%) [27], Hosana hospital (28.3%) [28], and Gondar Northern Ethiopia 28.8% [29].

However, this finding was much lower than the findings reported in studies conducted in Mumbai (69%) [30] and Brazil (72%) [31]. This might be due to the variation in the socioeconomic status and access to information regarding dual contraceptive methods.

Moreover, the current study reported a higher prevalence of dual contraceptive utilization compared with studies conducted in India (23%) [32], South Africa (6.8%) [33] and Tigray region, Ethiopia (21.6%) [34]. This might be due to the variation in the linkage of family planning services at service delivery points with ART services. Because, in this study, only 37% of the respondents have assessed family planning at ART (Table 3). This finding implies the need of strengthening the implementation of existing strategies that focus on promotion of dual contraceptive utilization to achieve UNAIDS proposed targets for HIV for 2030 for low and middle income countries (New infections among adults should be reduced to 200,000) and sustainable development goal-3 aims by 2030, end the epidemics of AIDS [35].

The odds of utilizing dual contraceptive methods were higher for individuals who disclosed their HIV sero-status to the community. This might be due to the fact that disclosure increases patient attendance at health facility appointments [16, 36]. Moreover, this finding implies that the need for avocations on the importance of disclosing one's HIV sero-status to the community through mass media. The concerned bodies should strengthen the existing strategies to increase the uptake of disclosure to community in turn to decrease stigma and increase dual contraceptive utilization [37].

The odds of utilizing dual contraceptive methods were higher for study participants who disclosed their HIV sero-status than for their counterparts. This finding agrees with those reported in studies conducted in north Ethiopia [38] and Kenya [39]. In contrast, the study conducted in West Zone hospitals in Ethiopia reported that individuals who did not disclose their HIV status were more likely to use dual contraceptive methods compared with their counterparts [20]. This might be due to lack of knowledge regarding HIV transmission. This finding implies that attention should be given to awareness creation on the benefits of disclosure.

Discussion with husband on family planning increases the likelihood of dual contraceptive utilization for that individual 5.8 times compared with their counterparts. This finding is consistent with those reported in studies conducted in Gondar [14] and south west Ethiopia [17]. Moreover, WHO recommends male involvement, which is one of the health promotion strategies that can increase the uptake of maternal and child health services. Thus, the finding of this study implies the need for strengthening the existing strategies to improve dual contraceptive utilization [40].

Study participants who had history of STIs were 3.2 times more likely to use dual contraceptive methods compared with their counterparts. This might be due to its dual effect. This finding agrees with that reported in a study conducted in Nigeria [41]. This showed that the likelihood of contracting STIs is less among individuals who used dual contraception. This finding implies that the need for strengthening of integration of STI care with family planning service provision.

The HIV sero-status of sexual partners is associated with dual contraceptive utilization. The odds of utilizing dual contraceptive methods were 3.7 times higher for study participants whose partners’ HIV sero-status was positive. This finding contradicts with the study conducted in Mekele, which reported that having HIV-negative partners was positively associated with the use of dual contraceptives by women living with HIV [38]. This finding implies that in addition to the prevention of HIV transmission to sexual partners with HIV-negative sero-status, the policy makers and concerned bodies should focus on the strategies that reduce the acquisition of new strains of HIV.

The current study also found an association between fertility desire and dual contraceptive method utilization. The odd of utilizing dual contraceptive methods was 4.3 times higher for participants who had no desire for fertility compared with that for their counterparts. This discovery demonstrated that there was no need for an unwanted desire for fertility due to the possibility of pregnancy and STI complications [42]. This finding is similar to that reported in a study conducted at Fitche hospital, where it was revealed that individuals who had no fertility desire were more likely to use dual contraceptive methods [27].

Individuals who were sexually active were more likely to use dual contraceptive methods compared with their counterparts. This finding is similar to that reported in a study conducted in Fitche hospital [27]. This indicated that knowledge and confidence developed by the study participants due to availability of sexual education. Moreover, this finding implies the need of sexual education for those participants who were not sexually active.

### 4.1 Strength and limitations of the study

There was limited evidence on the magnitude and determinants of dual contraceptive utilization in this study area. Thus, it adds inputs at the local level. It also improves family planning service implementation at health facilities. Because this study is cross-sectional, no cause and effect relationship was reported. In addition, social desirability bias could be another limitation. Moreover, this study is institutional based; therefore, the results of this study cannot be generalized to mothers attending health institutions outside the study area and at the community level.

## 5 Conclusion

The magnitude of dual contraceptive utilization was found to be low in this study compared with the findings in a number of other studies. The main predictors of dual contraceptive utilization were fertility desire, being sexually active, HIV sero-status of the partners, discussion with the husband on dual contraceptive methods, HIV sero-status disclosure, and having of a history of STIs. The majority of the study participants had used condoms plus injectables followed by condoms plus implants, condoms plus pills and condoms plus IUCDs.

This finding implies the need for the integration of F/P with STI care and ART. Moreover, the concerned bodies and policy makers should focus on developing strategies that promote sexual education and help in reducing the acquisition of new strains of HIV among HIV-positive partners.

## 6 What is already known in this topic?

Dual contraceptive utilization is important to prevent acquiring new HIV strain.

Utilization of dual contraceptive methods is depend on commitment of both sexual partners.

### 6.1 What this study adds?

This study adds the level of dual contraceptive utilization in this Wolaita zone.

This study also adds the importance of integrating HIV care with sexually transmitted disease and family planning.

The finding of this study implies that the concerned bodies should gave attention to developing strategies that promote sexual education to reduce acquiring new strains of HIV.

## Data sharing statement

The data used to support the findings of this study are available from the corresponding author upon request, email address: derehaile2010@gmail.com.

## Declarations

### Author contribution statement

Berhanu Lagebo and Dereje Haile: Conceived and designed the experiments; Performed the experiments; Analyzed and interpreted the data; Wrote the paper.

### Funding statement

This research did not receive any specific grant from funding agencies in the public, commercial, or not-for-profit sectors.

### Data availability statement

Data will be made available on request.

### Declaration of interests statement

The authors declare no conflict of interest.

### Additional information

No additional information is available for this paper.

## References

[1] WHO., G. (15 Nov 2019).

[2] Ethiopia., F.D.R.o. (2012).

[3] FHAPCO, F.H.A.P.a.C.O. (November 2018).

[4] USAIDS (2018).

[5] AIDS, G.i.a.e.o.H.a. (19 December 2018). Prevention OF mother-to-child transmission (PMTCT) OF HIV. Avert.

[6] CO., A. (2011). *Contraception in the Context of HIV/AIDS:.* A review. Afr. J. Reprod. Health.

[7] Team., I.-a.T. (2015). https://www.k4health.org/.

[8] Tesfaye Regassa Feyissa M.L.H. (2019). Alemu Sufa Melka & Deborah Loxton, unintended pregnancy in women living with HIV in sub-Saharan Africa: a systematic review and meta-analysis. AIDS Behav..

[9] O., C. (2012). Contraception in the context of HIV/AIDS. Afr. J. Reprod. Health.

[10] Jenny AH A.D. (2015).

[11] Wilson TE K.L., Walter E., Fernandez I., Ethier K. g (2003). Dual contraceptive method use for pregnancy and disease prevention among HIV infected and HIV uninfected women: the importance of an event-level focus for promoting safer sexual behaviors. PubMed.

[12] O, C. (Sepetmber 2011). Contraception in the context of HIV. Afr. J. Reprod. Health.

[13] Dereje Bayissa Demissie T.G.a.G.A. (2015). Dual contraceptive utilization and associated factors among people living with HIV attending ART clinic in Fitche hospital, Ethiopia. SM J. Commun. Med. SM Group.

[14] Mebratu Mitiku Reta G.A.T.a.G.S. (2019). Prevalence of dual contraceptive use and associated factors among HIV positive women at University of Gondar Hospital Northwest Ethiopia. BMC Res. Notes.

[15] Dankoli R.S., Aliyu A.A., Nsubuga P., Nguku P., Ossai O.P., Tukur D., Abdullaziz M. (2014). HIV disclosure status and factors among adult HIV positive patients in a secondary health facility in North-Eastern Nigeria 2011. Pan Afr. Med. J..

[16] Alemayehu M., Aregay A., Kalayu A., Yebyo H. (2014). HIV disclosure to sexual partner and associated factors among women attending ART clinic at Mekelle hospital, Northern Ethiopia. BMC Publ. Health.

[17] Meseret W Mariam Erashi1, F.Y.T.a.T.T.B. (2015). Dual-contraceptive method utilization and associated factors among HIV positive women attending Art clinic in Gebretsadik Shawo hospital SNNPR, south west Ethiopia. J. Women's Health Care.

[18] Lawari Lucky O., Onyebuchi Azubulike K., Iyoke C.A. (2014). Dual method use for protection of pregnancy and disease prevention among HIV infecte women in south east Nigeria. BMC Women Health.

[19] Munsakul W. (2016). Dual contraceptive method use and pregnancy intention among people living with HIV receiving HIV care at six hospitals in Thailand. Reprod. Health.

[20] Gudisa D.B.D.a.T. (2019).

[21] ICF., C.S.A.C.E.a. (2016).

[22] Berhane Y. (2013).

[23] WHO (2013 FEB 3).

[24] Warangkana Munsakul R.L. (2016). Dual contraceptive method use and pregnancy intention among people living with HIV receiving HIV care at six hospitalsn in Thailand.

[25] Ezugwu E.C. (2014). Contraceptive use among HIV-positive women in Enugu, southeast Nigeria. Int. J. Gynaecol. Obstet..

[26] Polisi A. (2014). Modern contraceptive utilization among female ART attendees in health facilities of Gimbie town, West Ethiopia. Reprod. Health.

[27] Demissie, D.B.T.G.G.A. (2015). Dual contraceptive utilization and associated factors among people living with HIV attending ART clinic in Fitche hospital,Ethiopia. Health Med. Nurs..

[28] Selamu Jifar T.B.H. (2017).

[29] al ., A.e. (2020). Dual contraception method utilization and associated factors among sexually active women on antiretroviral therapy in Gondar City, northwest, Ethiopia: a cross sectional study. BMC Wom. Health.

[30] Beena Joshi e.a. (2015). Contraceptive Use and unintended pregnancies among HIV-infected women in Mumbai. Indian J. Community Med..

[31] Kiyomi Tsuyuki R.M.B., de Araujo Pinho Adriana (2013).

[32] Chakrapani Venkatesan, Kershaw Trace, Shunmugam Murali, Newman Peter A., Cornman Deborah H., Dubrow Robert (2011). Prevalence of and Barriers to dual-contraceptive methods Use among married men and women living with HIV in India. Hindawi Publishing corporation Journal of sexually transmitted diseases. Infect. Dis. Obstet. Gynecol..

[33] Grimsrud, C.M.i.M.R.M.S.G.A. (2007).

[34] Berhane Y. (2013).

[35] WHO, U., UNICEF (2015).

[36] Getenet Dessie F.W., Mulugeta Henok, Dessalegn Amare, Jara Dube, Tesema Leshargie Cheru, Negesse Ayenew, Rayasam Swati, Burrowes Sahai (2019). The effect of disclosure on adherence to antiretroviral therapy among adults living with HIV in Ethiopia: a systematic review and meta-analysis. BMC Infect. Dis..

[37] Tamirat Melis Y.F., Lemma Lire (2020).

[38] Solomon Weldemariam Gebrehiwot G.A.A., Robles Carmen C., Mehretie Adinew Yohannes (2017). Utilization of dual contraception method among reproductive age women on antiretroviral therapy in selected public hospitals of Northern Ethiopia. BioMed Cent..

[39] Mideva Mulongo Agnes, Wekesa Lihana Raphael, Githuku Jane, Gura Zeinab, Simon Karanja (2017).

[40] WHO (2015).

[41] etal, L. (2014). Dual method use for protection of pregnancy and disease prevention among HIV-infected women in South East Nigeria. BMC Wom. Health.

[42] Reitter A., AUS, Linde R., Königs C., Knecht G., E Herrmann R Schlößer, Louwen F., Haberl A. (2014).

